# Anti-Pollutant Activity of *Porphyra yezoensis* Water Extract and Its Active Compound, Porphyra 334, against Urban Particulate Matter-Induced Keratinocyte Cell Damage

**DOI:** 10.3390/md21020121

**Published:** 2023-02-13

**Authors:** Seoyoung Choi, Jeong Hun Lee, Sae Woong Oh, Eunbi Yu, Kitae Kwon, Sung Joo Jang, Dong Sun Shin, Sang Hyun Moh, Jongsung Lee

**Affiliations:** 1 Molecular Dermatology Laboratory, Department of Integrative Biotechnology, College of Biotechnology and Bioengineering, Sungkyunkwan University, Suwon City 16419, Gyunggi Do, Republic of Korea; 2 Anti-Aging Research Institute of BIO-FD&C Co., Ltd., Incheon 460810, Republic of Korea

**Keywords:** urban particulate matter (UPM), transient receptor potential vanilloid 1 (TRPV1), aryl hydrocarbon receptor (AhR), reactive oxygen species (ROS), porphyra 334

## Abstract

Urban particulate matter (UPM) causes skin aging and inflammatory reactions by influencing skin cells through the aryl hydrocarbon receptor (AhR) signaling pathway. *Porphyra yezoensis* (also known as *Pyropia yezoensis*), a red alga belonging to the Bangiaceae family, is an edible red seaweed. Here, we examined the anti-pollutant effect of *P. yezoensis* water extract. While UPM treatment induced xenobiotic response element (XRE) promoter luciferase activity, *P. yezoensis* water extract reduced UPM-induced XRE activity. Next, we isolated an active compound from *P. yezoensis* and identified it as porphyra 334. Similar to the *P. yezoensis* water extract, porphyra 334 attenuated UPM-induced XRE activity. Moreover, although UPM augmented AhR nuclear translocation, which led to an increase in cytochrome P450 1A1 (CYP1A1) mRNA levels, these effects were reduced by porphyra 334. Moreover, UPM induced the production of reactive oxygen species (ROS) and reduced cell proliferation. These effects were attenuated in response to porphyra 334 treatment. Furthermore, our results revealed that the increased ROS levels induced by UPM treatment induced transient receptor potential vanilloid 1 (TRPV1) activity, which is related to skin aging and inflammatory responses. However, porphyra 334 treatment reduced this reaction by inhibiting ROS production induced by CYP1A1 activation. This indicates that porphyra 334, an active compound of *P. yezoensis*, attenuates UP-induced cell damage by inhibiting AhR-induced ROS production, which results in a reduction in TRPV1 activation, leading to cell proliferation. This also suggests that porphyra 334 could protect the epidermis from harmful pollutants.

## 1. Introduction

Particulate matter (PM) is a mixture of particles suspended in the atmosphere. PM is produced from factories, power plants, waste incinerators, automobiles, construction activities, fires, and unmanaged windblown dust [1]. Metals, organic compounds, materials with a biological origin, ions, and the carbon core of the particle are the major constituents of PM [2]. Particularly in urban areas, urban particulate matter (UPM) has become a significant risk factor for human health. UPM has been demonstrated to increase the risk of pulmonary and cardiovascular diseases, and cancer [3,4,5,6]. Numerous epidemiological studies indicate that PM may pass through the skin’s protective layer and increase the levels of reactive oxygen species (ROS), which worsens skin inflammation [7,8,9,10]. Other epidemiological data also suggest that pollutants may worsen atopic dermatitis symptoms in children, particularly the sense of itching, and may also be the source of elevated urinary ROS in atopic dermatitis patients [7,8,9,11].

UPM is a well-known activator of the latent transcription factor aryl hydrocarbon receptor (AhR), which controls cell inflammation, proliferation, and apoptosis [12]. UPM activates the AhR signaling pathway, which increases the production of proinflammatory cytokines and ROS [13]. AhR translocates into the nucleus as a result of the binding of UPM to AhR in the cytoplasm. In the nucleus, AhR activated by UPM interacts with the AhR nuclear translocator (ARNT), which activates the expression of its target gene, cytochrome P450 1A1 (CYP1A1), by interacting with the xenobiotic response element (XRE) [13].

Transient receptor potential vanilloid 1 (TRPV 1), a calcium-permeable, non-selective cation channel, is widely expressed in primary sensory neurons and mesenchymal and epithelial human skin cell types, including mast cells, glial cells, bronchial epithelial cells, uroepithelial cells, and keratinocytes [14,15]. A variety of unpleasant stimuli, such as pH, heat, endogenous lipid derivatives, and exogenous chemicals, which can produce pain, inflammation, and hyperalgesia, can activate the polymodal sensory transducer TRPV1 [16]. Furthermore, ROS may directly increase plasma membrane permeability to calcium ions by activating TRPV1 cation channels following conformational changes in this essential membrane protein [17,18].

Antioxidants are abundant in seaweed and have drawn significant interest from researchers, consumers, and the food industry [19]. These macroalgae adapt special metabolic mechanisms to survive in harsh environments, which leads to the production of a variety of secondary metabolites, some of which are antioxidant peptides [20]. The red seaweed *Porphyra* is also referred to as “nori” in popular culture. One of the most lucrative macroalgal species in the world, aquaculture accounts for most of its production [21]. *Porphyra yezoensis* is abundant in eastern Asian regions [22]. Porphyra 334, one of the active compounds of *P. yezoensis*, and recent findings have demonstrated that extracts of algae such as *Porphyra* 334 can protect human fibroblasts from UV-induced photodamage [23].

In this study, we examined the roles of the AhR and TRPV1 signaling pathways as important mechanisms of UPM-mediated adverse health effects, especially regarding the generation of oxidative stress. Additionally, we investigated the effect of *P*. *yezoensis* extract and its active compound, porphyra 334, on the UPM-induced cell damage in human keratinocytes.

## 2. Results

### 2.1. Porphyra yezoensis Extract Reduces ROS Production Induced by UPM through Inhibiting XRE Promoter Activity

**Figure 1:**
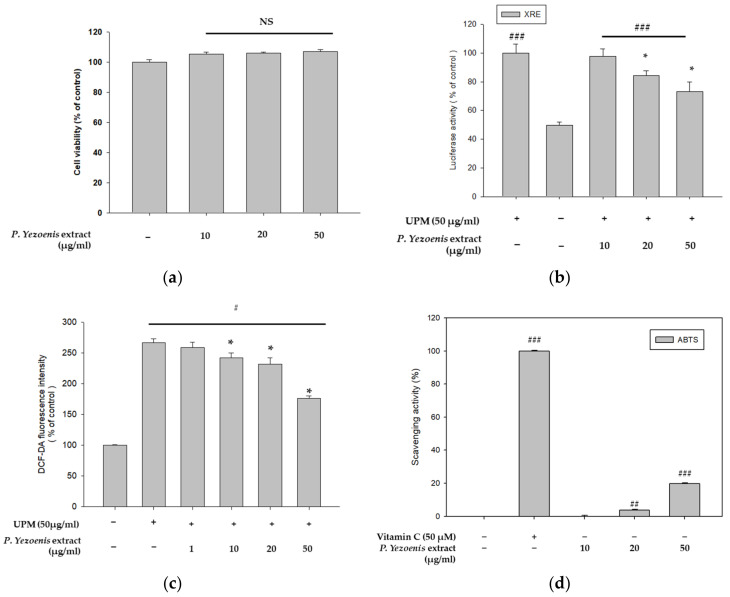
Effect of *P. yezoensis* extract on XRE promoter activity induced by UPM in human keratinocytes. (**a**) HaCaT cells were treated with *P. yezoensis* extract (10, 20, 50 μg/mL) for 24 h and cell viability was measured using a CCK-8 assay. (**b**) After being transfected with the XRE-luc reporter, HaCaT cells were cotreated for 24 h with UPM (50 μg/mL) and *P*. *yezoensis* extract (10, 20, 50 μg/mL). The cells were next examined using a luciferase reporter assay, and the measurements were normalized by β-galactosidase activity. (**c**) ROS levels were quantified using 2′7′-dichlorofluorescein diacetate. (**d**) ABTS radical scavenging activity of *P. yezoensis* extract was measured. (# *p* < 0.05: vs. control, ## *p* < 0.01: vs. control, ### *p* < 0.001: vs. control, * *p* < 0.05: vs. UPM, NS: not significant vs. control) Porphyra extract: *P*. *yezoensis* extract.

The cytotoxicity of *P. yezoensis* extract was first measured using the CCK-8 assay to select their treatment concentrations. *P*. *yezoensis* extract showed no cytotoxicity at any of the concentrations tested (Figure 1a). UPM was reported to increase intracellular ROS levels by activating XRE promoter activity [24]. Therefore, we analyzed XRE promoter activity in cells treated with UPM and with or without *P*. *yezoensis* extract (10, 20, 50 μg/mL) for 24 h. UPM-treated cells showed increased XRE promoter activity, while cells cotreated with *P*. yezoensis extract attenuated this effect (Figure 1b). Next, we conducted a DCF-DA cellular ROS detection assay to investigate the involvement of *P*. yezoensis extract in ROS production induced by UPM in HaCaT cells. Since 50 μg/mL of UPM showed the highest ROS production, the UPM concentration was set to 50 μg/mL in the ROS production assay (Figure S1). ROS levels were increased by treatment with UPM, while cells co-treated with *P*. *yezoensis* extract reduced their levels in a dose-dependent manner (Figure 1c). Furthermore, we identified the effect of *P*. *yezoensis* extract on radical-scavenging activity. *P*. *yezoensis* extract showed a significant effect at the highest concentration (Figure 1d). These data indicate that *P*. *yezoensis* extract has anti-pollutant and antioxidant activities in human keratinocytes. 

### 2.2. Preparation of P. yezoensis Extract and Porphyra 334 from Laver

**Figure 2:**
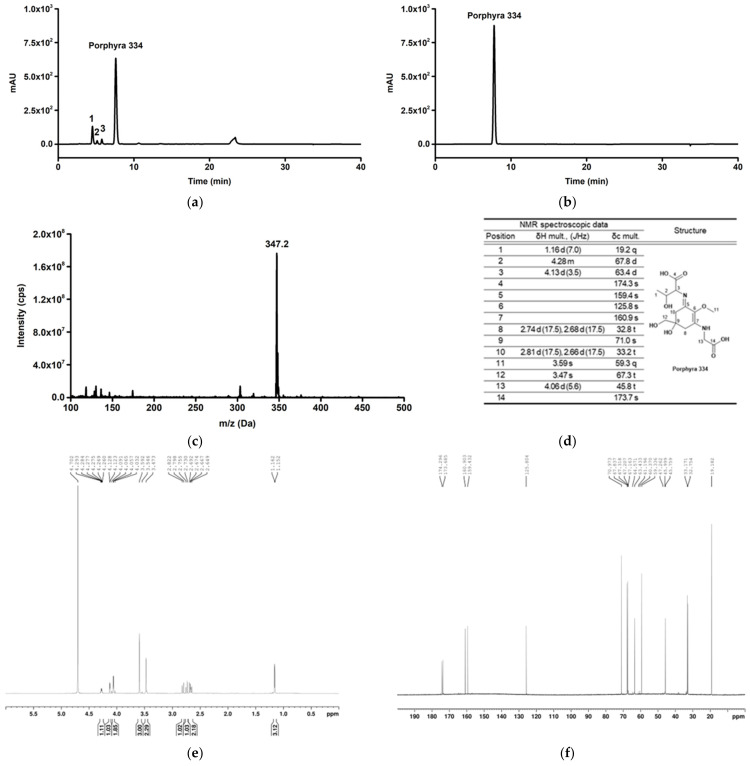
*P. yezoensis* extract and porphyra 334 analysis. (**a**,**b**) A porphyra 334 peak with retention time of 7.7 min appeared in both samples. (**a**) The result of HPLC analysis of porphyra extract. (**b**) The result of HPLC analysis of purified porphyra 334. (**c**–**f**) The molecular weight and structure of purified porphyra 334 were confirmed through MS and NMR. (**c**) [M + H]^+^ at m/z 347.2 observed in the mass spectrum. (**d**) The positions of carbons matched with each chemical shift (δH and δC) in spectroscopic data of NMR. (**e**) ^1^H Spectrum of purified porphyra 334. (**f**) ^13^C Spectrum of purified porphyra 334.

In previous experiments, we found that *P*. *yezoensis* extract showed antioxidant and anti-pollutant activities. Therefore, we examined the active components of *P*. *yezoensis* extract and its chemical structure. In this study, we found several compounds including porphyra 334 and screened their activity using a DCF-DA cellular ROS detection assay. Since porphyra 334 showed the highest antioxidant activity (Figure S2), we focused on it. First, 75 g of *P*. *yezoensis* extract powder was obtained from 500 g of laver, and 1.1 g of porphyra 334 was subsequently obtained through purification and freeze-drying. The HPLC chromatograms of the *P*. *yezoensis* extract and purified porphyra 334 at 330 nm UV are shown in Figure 2a,b. As a result of analysis using HPLC, a main peak (porphyra 334) with a retention time of 7.7 min appeared in the chromatogram. Other minor peaks were observed in the *P*. *yezoensis* extract, and only one peak was observed in purified porphyra 334. The area ratio of the main peak of purified porphyra 334 was 99.9% of the total peak area. After HPLC analysis, the substances were analyzed using mass spectrometry. Sample injection into the instrument was carried out by the syringe infusion method (flow rate of 20.0 µL/min), and the [M + H]+ of m/z 347.2 (the protonated molecular ion of porphyra 334) was observed in the spectrum (Figure 2c). The molecular structure of porphyra 334 was confirmed by ^1^H and ^13^C NMR spectra at 700 and 175 MHz, respectively. Each structural position of the carbon corresponding to the position and number of each chemical shift (δH, δC) was identified using spectroscopic data (Figure 2d–f). A quantitative test of porphyra 334 contained in *P. yezoensis* extract was performed using the purified porphyra 334 as an analytical standard compound; 1 g of the extract contained 15.1 ± 0.04 mg of porphyra 334.

### 2.3. Porphyra 334 Induces Cell Proliferation by Inhibiting ROS Production Induced by UPM in Human Keratinocytes

**Figure 3:**
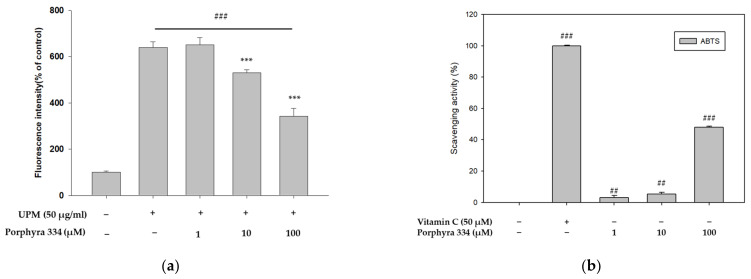

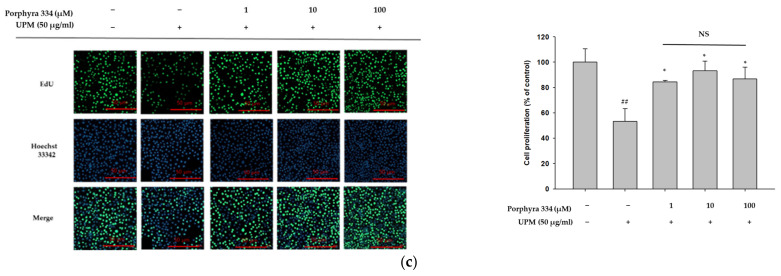
Effect of porphyra 334 on cell proliferation and ROS production induced by UPM in human keratinocytes. (**a**) HaCaT cells were cotreated with porphyra 334 (1, 10, 100 μm) and UPM (50 μg/mL) for 24 h. ROS production was measured using 2′7′-dichlorofluorescein diacetate. (**b**) ABTS radical scavenging activity of porphyra 334 were detected. (**c**) Cell proliferation was measured using an EdU incorporation assay after being treated with porphyra 334 and UPM for 24 h. (## *p* < 0.01: vs. control, ### *p* < 0.001: vs. control, * *p* < 0.05: vs. UPM, *** *p* < 0.001: vs. UPM, NS: not significant vs. control).

In previous experiments, *P. yezoensis* extract was shown to lower ROS production in cells treated with UPM. We next determined the anti-pollutant effect of porphyra 334, which is an active compound in *P. yezoensis* extract. The cytotoxicity of porphyra 334 was first measured using the CCK-8 assay to select their treatment concentrations. Porphyra 334 showed no cytotoxicity at any of the concentrations tested (Figure S3). We first examined the antioxidant activity of porphyra 334 in UPM-treated cells. Porphyra 334 attenuated the cellular ROS levels induced by UPM in a dose-dependent manner (Figure 3a). Since porphyra 334 exhibited antioxidant activity, we performed an ABTS assay to examine its radical scavenging activity. Porphyra 334 did not show greater radical scavenging activity than vitamin C, which was used as the positive control (Figure 3b). However, it had a significant effect on radical scavenging activity (Figure 3b). We further investigated the effect of porphyra 334 on cell proliferation by using the EdU cell proliferation assay. EdU (5-ethynyl-2′-deoxyuridine) is a thymidine nucleoside analog and is integrated into DNA during active DNA synthesis. Therefore, incorporating EdU allows us to detect cell proliferation. UPM-treated cells showed reduced proliferation, whereas cells cotreated with porphyra 334 reversed this effect (Figure 3c). These results indicated that porphyra 334 reduced UPM-induced cell damage by attenuating cellular ROS production. 

### 2.4. Porphyra 334 Exerts Anti-Pollutant Effects by Reducing XRE Promoter Activity

**Figure 4:**
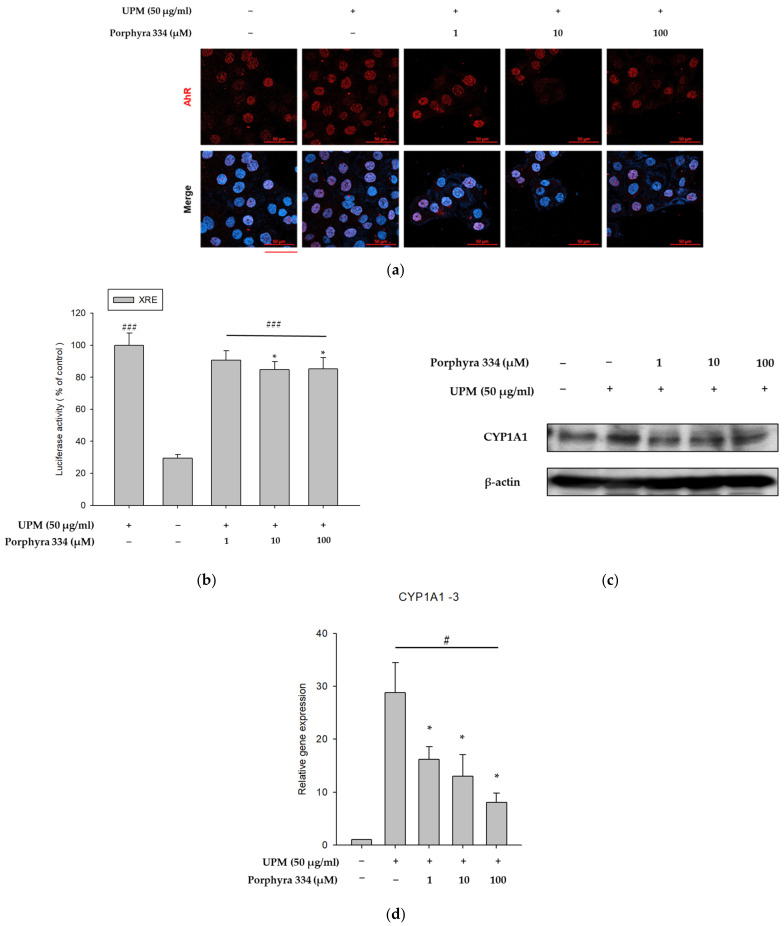
Effect of porphyra 334 on AhR signaling induced by UPM. (**a**) The fluorescence intensity of AhR and DAPI were measured in response to both UPM and porphyra 334 treatment using immunofluorescence assay. (**b**) HaCaT cells were cotreated for 24 h with UPM (50 μg/mL) and porphyra 334 (1, 10, 100 μm) after being transfected with the XRE-luc reporter. The measurements were then normalized by β -galactosidase activity. (**c**) Western blotting was used to assess the expression of the CYP1A1 protein. β-actin was used as the control for whole cell lysates. (**d**) qRT-PCR was used to determine the levels of CYP1A1 mRNA level. Glyceraldehyde 3-phosphate dehydrogenase (GAPDH) was used as the control. (# *p* < 0.05: vs. control, ### *p* < 0.001: vs. control, * *p* < 0.05: vs. UPM).

UPM is known to induce ROS production by activating AhR and its downstream signals [25]. Therefore, we investigated the effects of porphyra 334 on UPM-induced AhR signaling by analyzing AhR nuclear translocation, cytochrome P450 1A1 (CYP1A1) expression, and XRE-luciferase activity. In the experiment for nuclear translocation of AhR, even in the absence of UPM, some AhR was found in the nucleus, but a large amount of AhR was expressed in the nucleus by UPM treatment, indicating that AhR moved to the nucleus by UPM. As shown in Figure 4a, UPM treatment induced nuclear translocation of AhR, whereas co-treatment with porphyra 334 reduced its effect. Furthermore, porphyra 334 reduced the UPM-induced XRE-luciferase activity (Figure 4b). Additionally, whereas UPM increased the protein and mRNA levels of CYP1A1, porphyra 334 inhibited the UPM-induced elevation of CYP1A1 (Figure 4c,d). The densitometric analysis for Figure 4c was shown in Figure S4. These findings indicate that porphyra 334 exerts anti-pollutant effects by inhibiting AhR signaling molecules.

### 2.5. Porphyra 334 Inhibits the Activation of TRPV1 and Calcium Influx Induced by UPM

**Figure 5:**
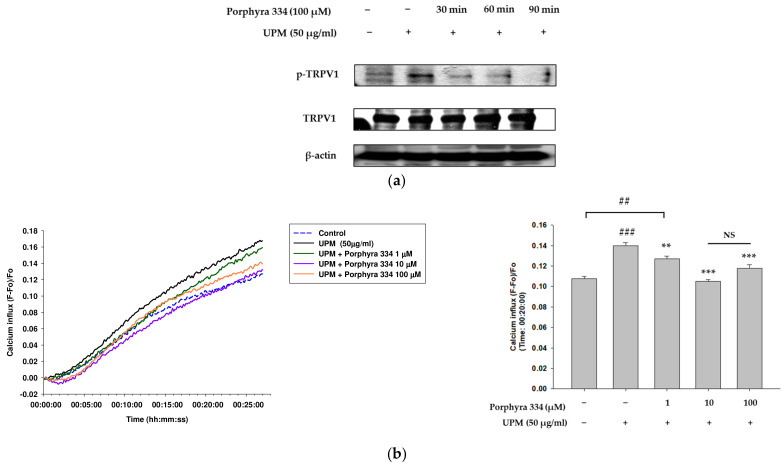
Effect of porphyra 334 on TRPV1 signaling induced by UPM. (**a**) In experiments in which HaCaT cells were treated with UPM (50 μg/mL) for 90 min, porphyra 334 (100 μm) was co-treated for 30 min, 60 min, and 90 min, respectively. After 90 min, the cells were harvested and the phosphorylated form of TRPV1 was detected using Western blot analysis. (**b**) The Fluo-4 NW assay was used to assess calcium influx into HaCaT cells. Calcium influx was measured after 20 min incubation of treated samples. (## *p* < 0.01: vs. control, ### *p* < 0.001: vs. control, ** *p* < 0.01: vs. UPM, *** *p* < 0.001: vs. UPM, NS: not significant vs. control).

In our previous experiments, we determined that UPM increases ROS production by inducing the nuclear translocation of AhR and its downstream signals. ROS induces TRPV1 activation through a conformational change [17,18]. Therefore, we next identified the effect of porphyra 334 on TRPV1 signaling induced by UPM. Western blotting was used to analyze the phosphorylation of TRPV1. UPM increased TRPV1 phosphorylation, while co-treatment with porphyra 334 lowered its effect (Figure 5a). The densitometric analysis for Figure 5a was shown in Figure S5. Furthermore, we examined the calcium influx levels induced by TRPV1 activation and the effects of UPM and porphyra 334. UPM-treated cells showed increased calcium influx, whereas porphyra 334 with UPM treatment decreased calcium levels in human keratinocytes (Figure 5b). These data indicate that porphyra 334 regulates TRPV1 activation induced by UPM.

## 3. Discussion

In this study, for the first time, we demonstrated the antagonizing effects of *Porphyra yezoensis* extract and its active compound, porphyra 334 against urban particulate matter (UPM)-induced keratinocyte cell damage. Porphyra 334 suppressed AhR signaling, and ROS production induced by UPM in HaCaT cells. This study showed that the inhibitory effects of porphyra 334 on UPM were mediated by suppressing both nuclear translocations of AhR and TRPV1 activation. This study suggests that porphyra 334 could protect the skin from harmful UPM effects, contributing to the development of therapeutic agents against skin diseases using marine materials.

The epidermis, which is the outermost layer of the skin, is in direct contact with environmental stressors such as air pollution [26,27]. UPM can cause aberrant skin conditions such as allergic responses, aging, and slow wound healing. Keratinocytes are essential for maintaining the skin’s barrier function because they make up 95% of the epidermis in human skin. Therefore, in this study, we investigated the mechanism of action of the anti-pollutant effect of porphyra 334. In addition, the concentration of UPM used in the study was 50 μg/mL, which was known to cause skin barrier dysfunction and has been established in HaCaT cells as a UPM treatment model [28].

We first examined the anti-pollutant effects of *P. yezoensis* extract on human keratinocytes. *P. yezoensis* is a seaweed known as a marine medicinal herb that is used in Korea, Japan, and China [19,21]. It is well known for its antioxidant and anti-inflammatory effects [20,23,29]. We found that porphyra extract exhibits antioxidant activity by reducing UPM-induced XRE promoter activity in human keratinocytes. Therefore, we next identified the specific mechanism of action of the porphyra extract using its active compound, porphyra 334.

Porphyra 334 reduced the elevation in cellular ROS levels induced by UPM. Moreover, UPM-induced AhR signaling was attenuated by porphyra 334 treatment. Porphyra 334 inhibited AhR nuclear translocation and reduced XRE promoter activity and its target gene, CYP1A1. UPM-induced CYP1A1 expression and mRNA levels were reduced by porphyra 334 treatment. These data indicate that porphyra 334 exerts antioxidant activity by inhibiting AhR signaling.

The regulation of several symptoms, including pain, inflammation, hyperalgesia, and skin irritation, depends heavily on the expression and activation of TRPV1 [30,31]. ROS production induces TRPV1 activation, which elevates calcium influx and eventually increases proinflammatory cytokine levels. Our study showed that UPM-induced ROS levels increase TRPV1 activation, which leads to an increase in calcium levels. In contrast, the porphyra 334 treatment attenuated these effects. Additionally, cell proliferation, which was reduced by UPM treatment, was alleviated by porphyra 334 treatment. These results suggest that UPM-induced ROS levels increase TRPV1 activation, which leads to a decrease in cell proliferation while porphyra 334-treated cells reversed these effects.

The skin is in direct contact with environmental stresses such as UPM [26,27]. Numerous diseases have been linked to oxidative stress [32,33]. Therefore, finding antioxidant and cell-protective molecules has been a focus in the treatment of skin diseases. In this study, we determined the mechanism of action of porphyra 334 on its anti-pollutant activity. Porphyra 334 reduced AhR signaling molecules, which eventually decreased ROS production, leading to the inactivation of TRPV1 signaling. These results indicate that porphyra 334 reduces UPM-induced cell damage by attenuating cellular ROS production. However, we cannot also exclude the possibility that porphyra 334 exerts a protective activity against UPM through the regulation of ROS-independent cellular processes which is required to examine.

UPM can penetrate the human body through the oral and respiratory routes. Skin is another common pathway through which UPM enters the body. Alterations in the structure and function of the skin barrier by UPM can lead to various skin diseases. Our research suggests that although additional studies including clinical trials are needed, porphyra 334, an active compound in porphyra extract, may be used to treat skin conditions induced by UPM-induced oxidative stress.

## 4. Materials and Methods

### 4.1. Cell Culture and Materials

Human keratinocyte cell line HaCaT cells (American Type Culture Collection, Manassas, VA, USA) was grown in DMEM with 10% fetal bovine serum (FBS) and 1% antibiotics (penicillin/streptomycin) supplementation. The cultivated cells were maintained in a 37 °C incubator with a humidified 5% CO_2_ environment.

Antibodies against AhR (sc-133088) and CYP1A1 (sc-25304) were obtained from Santa Cruz Biotechnology (Dallas, TX, USA). Antibodies against p-TRPV1 (Ser502) (PA5-64860) were obtained from Invitrogen (Carlsbad, CA, USA). Antibodies against β-actin (A5316), anti-rabbit immunoglobulin G (IgG) (A0545), and anti-mouse IgG (A9044) were obtained from Sigma-Aldrich (Sigma-Aldrich, St. Louis, MO, USA).

### 4.2. Preparation of Porphyra yezoensis Extract and Porphyra 334 from Laver

The laver (*P. yezoensis*) used for the extraction of porphyra 334 was cultivated in Wando, South Korea, and dried after harvest. Water (20 L) was added to 500 g of dried laver and the mixture was extracted at 55 °C for 12 h. The extract was filtered through a 100-mesh filter, concentrated, and vacuum-dried to obtain the dried porphyra extract powder. The extract powder was dissolved in water and filtered through a membrane filter (pore size: 0.45 µm). Porphyra 334 was purified using a prep-LC (2525 binary pump with a photodiode array detector; Waters, Milford, MA, USA) using a prep-column (Gemini C18, 30 × 250 mm, 5 µm, Axia packed preparative column) and freeze-dried.

The prepared porphyra extract and porphyra 334 were analyzed using HPLC (1260 Infinity II system with a diode array detector, Agilent, Santa Clara, CA, USA) with a C18 column (Shim-pack GIST C18, 5 μm, 4.6 × 250 mm, Shimadzu, Japan), and a mixture of water and acetonitrile containing 0.1% trifluoroacetic acid was used as the eluent. The molecular weight of purified porphyra 334 was measured using mass spectrometry (AB SCIEX 3200 QTRAP MS/MS, Applied Biosystems, Foster City, CA, USA) and quantified in electrospray ionization-positive mode. The structure was confirmed by NMR (ADVANCE III 700 MHz NMR spectrometer, Bruker, Germany), and the powder was dissolved in D2O to measure the ^1^H and ^13^C NMR spectra.

### 4.3. Porphyra 334 and UPM Treatment

Porphyra 334 stock solution (1000 stock) was prepared in DMSO and stored at −20 °C before use. To obtain the final concentrations (1, 10, and 100 μm), porphyra 334 was further diluted with DMEM. Porphyra 334 was co-treated with urban particulate matter UPM (NIST1648A) for 24 h at a final concentration of 50 μg/mL.

### 4.4. ABTS Radical Scavenging Activity

The free radical scavenging abilities of *P. yezoensis* extract and porphyra 334 were investigated using the ABTS 2,2′-azino-bis(3-ethylbenzothiazoline-6-sulfonic acid) radical cation decolorization test. The interaction of 2.45 mM potassium persulfate (7727-21-1, Sigma-Aldrich, St. Louis, MO, USA) and 7 mM ABTS (30931-67-0, Sigma-Aldrich, St. Louis, MO, USA) in ultra-purified water (UPW) formed the ABTS+ cation radicals, which were then kept at 20 °C in the dark for 12 to 16 h before use. UPW was then added to the ABTS+ solution for dilution, resulting in an absorbance of 0.700 at 734 nm. *P. yezoensis* extract and porphyra 334 were added to the ABTS+ solution at various concentrations. The positive control was 50 μm vitamin C. Samples were tested at an absorbance of 734 nm. The following equation was used to determine the radical scavenging activity:

$$ABTS\;radical\;scavenging\;activity\;\left(\%\right)=\left(\frac{Absorbance\;of\;control-Absorbance\;of\;sample}{Absorbance\;of\;control\;}\right)\times 100$$

### 4.5. EdU Incorporation Assay

EdU incorporation assays for cell proliferation analysis were performed in accordance with the manufacturer’s instructions using the Click-iT^™^ EdU cell proliferation kit (A10044, Invitrogen, Waltham, MA, USA) for imaging. After adding 10 μm EdU to the cells cultured on glass coverslips, the coverslips were incubated for 12 h. The cells were then fixed for 15 min in 4% paraformaldehyde in PBS and permeabilized for 20 min at room temperature in 0.1% Triton X-100 and 0.01% Tween 20 after being rinsed three times in PBS. According to the manufacturer’s instructions, the cells were stained by incubating with the Click-iT^®^ reaction cocktail for 30 min in the dark. After three PBS washes, cells were counterstained with Hoechst 33342 (Invitrogen, Waltham, MA, USA). The cells were then placed on glass slides, coated in PBS, and studied using an LSM 700 laser-scanning confocal microscope equipped with a C-Apochromat 10× objective (Zeiss, Jena, Germany). The average fluorescence signal intensity was estimated after images were obtained with the same laser intensity. Images were analyzed using ZEN 2012 Blue (Zeiss, Jena, Germany) and ImageJ software 1.53e (National Institutes of Health, Bethesda, MD, USA).

### 4.6. Fluo-4 Ca^2+^ Influx Assay

The Ca^2+^ influx assay was performed using an Invitrogen Fluo-4 NW Ca^2+^ assay kit (F36206; Waltham, MA, USA). Cells were seeded and incubated in 96-well microplates with a clear bottom and a black wall for 24 h. Subsequently, the growth medium was removed and each well received a direct dose of Fluo-4 NW Ca^2+^ reagent loading solution. The dye-loading solution was applied to the cells for 30 min at 37 °C and for a further 30 min at room temperature in the dark. Following incubation, the solution was removed, and the cells were treated with assay buffer diluted porphyra 334 (1, 10, and 100 μm) and co-treated with UPM (50 μg/mL). Fluorescence was measured immediately following treatment using a microplate reader (Synergy HTX Multi-Mode Reader; Biotek, Winooski, VT, USA) at excitation/emission wavelengths of 494/516 nm.

### 4.7. DCF-DA Cellular ROS Detection Assay

A 2′,7′-dichlorofluorescein diacetate (DCF-DA) cellular ROS detection assay kit (ab113851; Abcam, Cambridge, UK) was used to assess ROS production according to the manufacturer’s instructions. Cells were cultured in 96-well microplates with clear bottoms and black walls. The cells were washed three times with PBS before staining with 20 μm DCF-DA in PBS for 30 min at room temperature under light-blocked conditions. The DCF-DA solution was discarded after the cells had been incubated, and PBS was used to wash the cells. Porphyra 334 (1, 10, and 100 μm) and UPM (50 μg/mL) dissolved in PBS were applied to cells together or separately. The fluorescence was measured at an Ex/Em ratio of 494/516 nm using a microplate reader.

### 4.8. Western Blot Analysis

HaCaT cells were grown on 60 mm plates and treated with porphyra 334 (1, 10, or μm) or UPM (50 μg/mL) for 24 h. Cells were harvested and centrifuged at 15,928× *g* for five min. RIPA lysis buffer (Thermo Fisher Scientific, Waltham, MA, USA) containing a phosphatase inhibitor cocktail was used to lyse the cells. The cells were lysed, and the supernatant was removed. The recovered proteins were separated using 7–10% SDS electrophoresis before being transferred to a PVDF membrane (162-0177, Bio-Rad, Hercules, CA, USA). The membrane was then blocked with a 2% bovine serum albumin (BSA) solution for 1 h before being incubated with primary antibodies overnight at 4 °C. The membrane was washed thrice with Tris-buffered saline containing Tween 20 before being probed with secondary antibodies for 1–2 h at room temperature. The blots were visualized using ECL western blotting reagents (170-5061, Bio-Rad, Hercules, CA, USA).

### 4.9. Luciferase Reporter Assay and β-Galactosidase Activity Assay

HaCaT cells were grown in 6-well plates and incubated in DMEM at 37 °C for 24 h. The cells were transiently co-transfected with 1 μg xenobiotic response element (XRE) (Stratagene, La Jolla, CA, USA) and 1 μg β-galactosidase plasmid. A total of 5 μg of polyethylenimine (PEI) (23966-2, Polysciences, Inc., Warrington, PA, USA) was used for transfection. After 4 h, fresh DMEM was added to the mixture in order to stabilize the cells. Transfected cells were treated for 24 h with or without porphyra 334 (1, 10, or 100 μm) and 50 μg/mL UPM. The cells were collected in phosphate-buffered saline (PBS), then centrifuged at 16,200× *g* for 5 min. Reporter lysis buffer (E3971; Promega, Madison, WI, USA) was used to lyse the centrifuged cells. After centrifuging the lysates at 12,000× *g* for 3 min at 4 °C, the supernatant was then transferred into 96-well plates. The luciferase and β-galactosidase activities in the supernatants were assessed according to the manufacturer’s instructions (Promega Corporation). XRE promoter activity was expressed as a ratio of firefly luciferase activity to β-galactosidase activity.

### 4.10. Statistical Analysis

All data were explained using the standard error of the mean and were confirmed in at least three independent experiments. Student’s *t*-test was used to examine the differences between the two groups. One-way analysis of variance and Tukey’s multiple comparison tests were performed using GraphPad Prism (5.0) (GraphPad, La Jolla, CA, USA) to compare the various groups. Statistical significance was set at *p* < 0.05.

## 5. Conclusions

**Figure 6:**
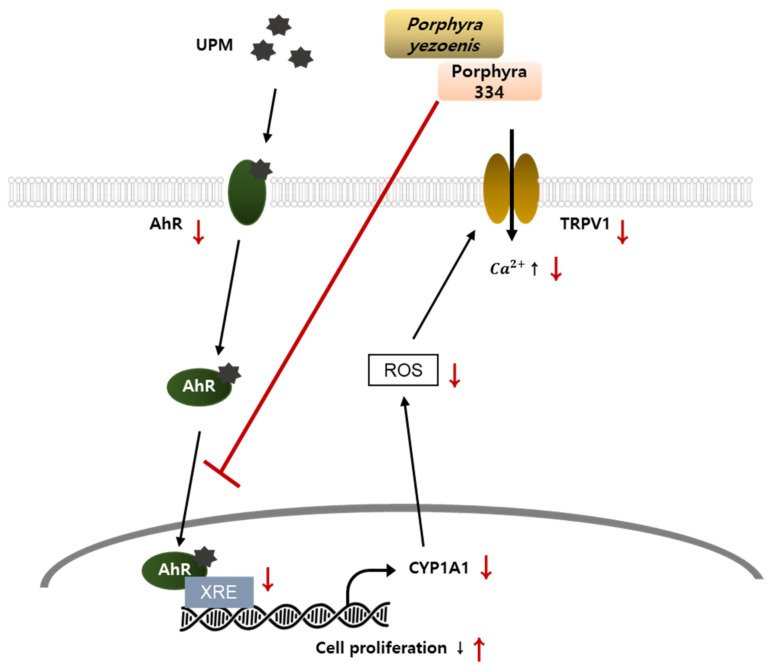
Action mechanism of porphyra 334 in the AhR and TRPV1-mediated signaling. Porphyra 334 inhibits UPM effects by inhibiting both AhR and TRPV1 signaling. Red line: action step of porphyra 334. A red arrow pointing up indicates activation, and a red arrow pointing down indicates inhibition.

This study demonstrated the antagonizing effects of *Porphyra yezoensis* extract and its active compound, porphyra 334 against Urban Particulate Matter (UPM)-induced keratinocyte cell damage. Porphyra 334 suppressed AhR signaling, as evidenced by the suppression of XRE reporter activation, CYP1A1 expression, and ROS production induced by UPM in HaCaT cells. This study showed that the inhibitory effects of porphyra 334 on UPM were mediated by suppressing both nuclear translocations of AhR and TRPV1 activation, as summarized in Figure 6. However, further studies are needed to define the AhR inhibitory effect of porphyra 334 as an antagonist effect. This study also suggests that porphyra 334 could protect the stratum corneum layer of the epidermis from harmful UPM.

## Data Availability

The data used to support the findings of this study are available from the corresponding author upon request.

## Supplementary Materials

The following supporting information can be downloaded at: https://www.mdpi.com/article/10.3390/md21020121/s1, Figure S1: DCF-DA analysis of UPM treatment in HaCaT cells. Figure S2: DCF-DA analysis of some compounds in HaCaT cells. Figure S3: Cell viability of porphyra 334. Figure S4: Western blot densitometry of CYP1A1. Figure S5: Western blot densitometry of p-TRPV1.
