# Supplementary material for: Anti-Pollutant Activity of Porphyra yezoensis Water Extract and Its Active Compound, Porphyra 334, against Urban Particulate Matter-Induced Keratinocyte Cell Damage
Source: Mar Drugs. 2023 Feb 13;21(2):121. doi: 10.3390/md21020121 (PMC9962167; doi:10.3390/md21020121)
Supplement: Supplementary file 1 [file marinedrugs-21-00121-s001.zip › marinedrugs-2161041-supplementary.pdf]

Supplemental Figure S1

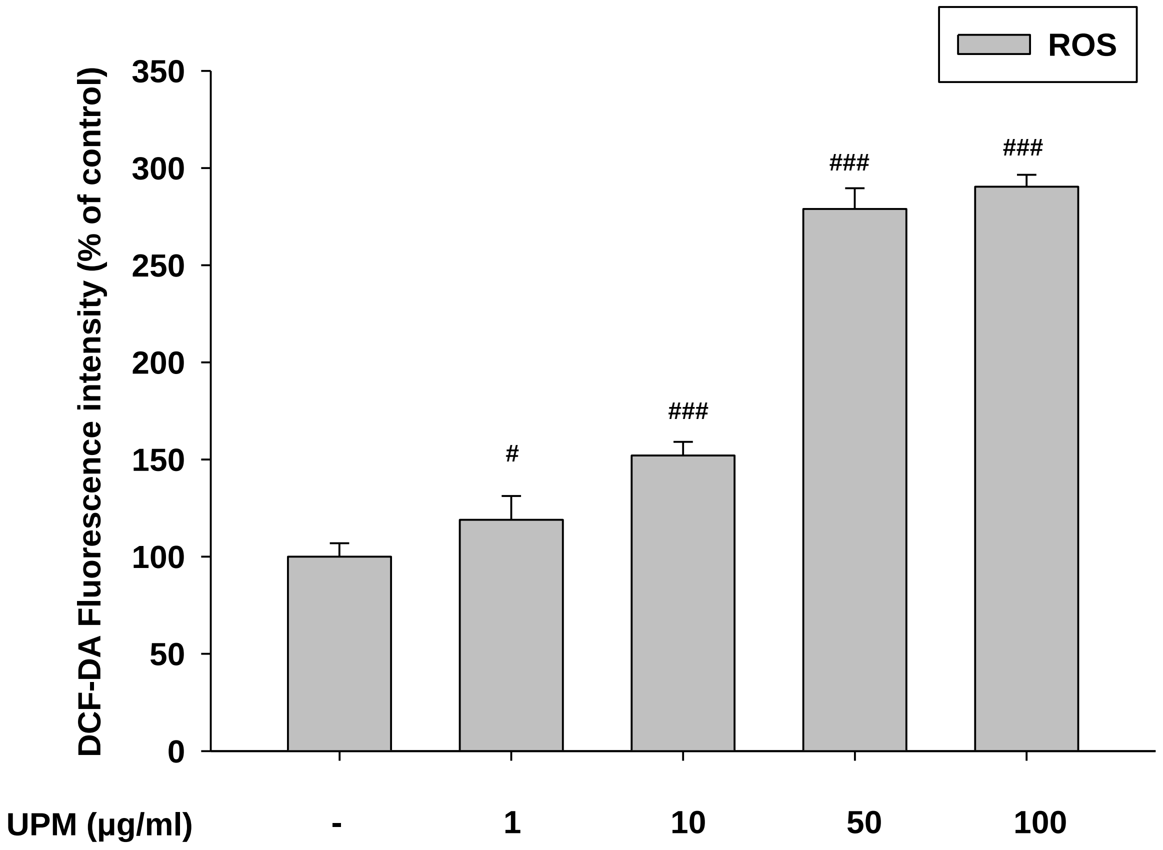

Supplemental Figure S1. DCF-DA analysis of UPM treatment in HaCaT cells. (#p < 0.05 : vs control,(###p < 0.001 : vs control)

Supplemental Figure S2

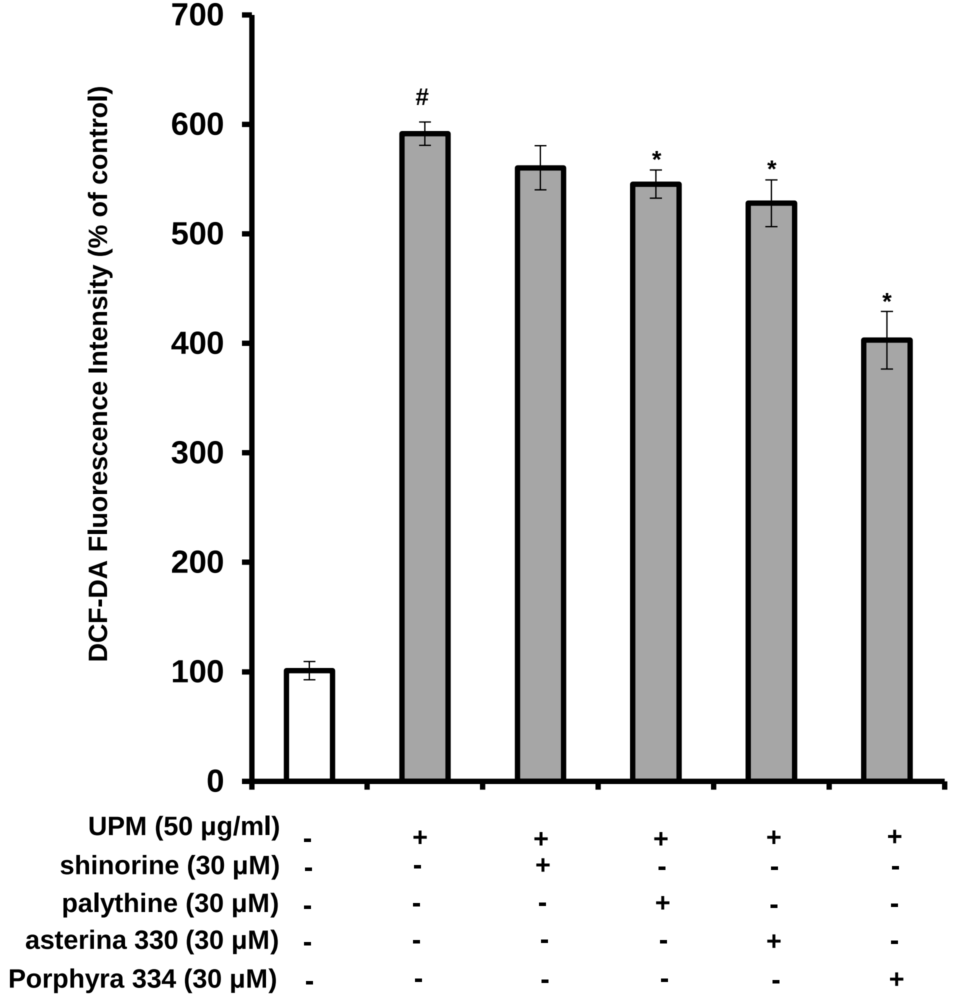

Supplemental Figure S2. DCF-DA analysis of compounds against UPM treatment in HaCaT cells.(#p < 0.05 : vs control, *p < 0.05 : vs UPM)

Supplemental Figure S3

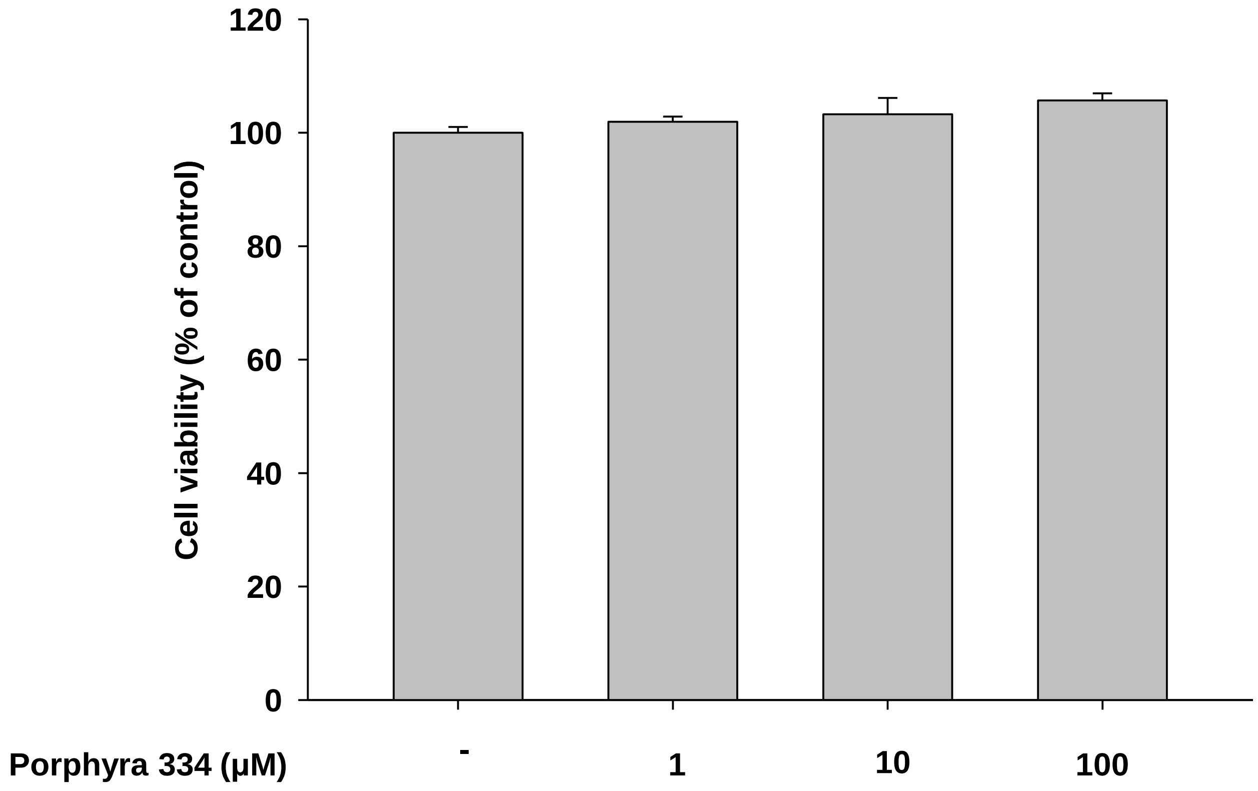

Supplemental Figure S3. Cell viability of porphyra 334

Supplemental Figure S4

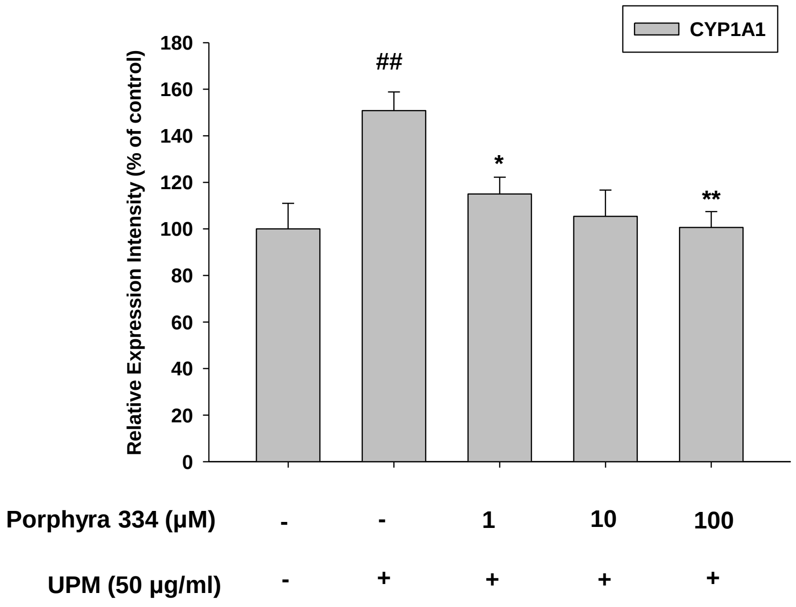

Supplemental Figure S4. Western blot densitometry of CYP1A1. HaCaT cells were cotreated for 24 h with UPM (50 μg/ml) and porphyra 334 (1, 10, 100 μM). Western blotting was used to assess the expression of the CYP1A1 protein. β-actin was used as the control for whole cell lysates.(##p < 0.01 : vs control, *p < 0.05 : vs UPM **p < 0.01 : vs UPM)

Supplemental Figure S5

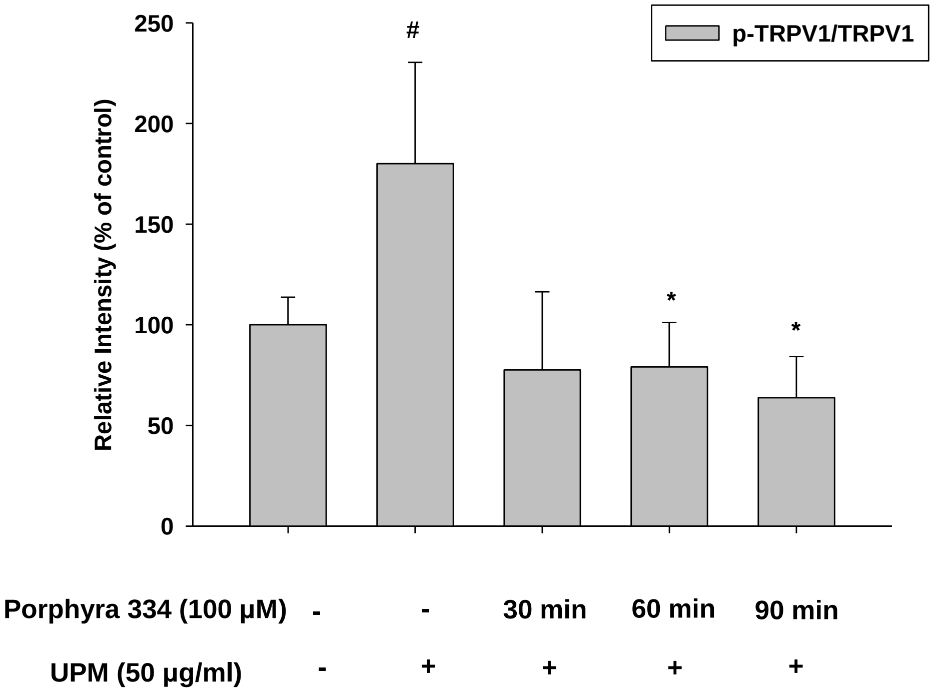

Supplemental Figure S5. Western blot densitometry of p-TRPV1. HaCaT cells were cotreated h with UPM (50 μg/ml) and porphyra 334 (1, 10, 100 μM) for 30, 60, 90 min. Western blotting was used to assess the expression of the p-TRPV1 protein. TRPV1 was used as the control for whole cell lysates. (#$p < 0.05$: vs control, *$p < 0.05$: vs UPM)
